# deepFPlearn ^+^: enhancing toxicity prediction across the chemical universe using graph neural networks

**DOI:** 10.1093/bioinformatics/btad713

**Published:** 2023-11-27

**Authors:** Kyriakos Soulios, Patrick Scheibe, Matthias Bernt, Jörg Hackermüller, Jana Schor

**Affiliations:** Department of Computation Biology, Helmholtz Centre for Environmental Research – UFZ, 04318 Leipzig, Germany; Department of Computer Science, Faculty of Mathematics and Computer Science, University of Leipzig, 04109 Leipzig, Germany; Department of Neurophysics, Max Planck Institute for Human Cognitive and Brain Sciences, 04103 Leipzig, Saxony, Germany; Department of Computation Biology, Helmholtz Centre for Environmental Research – UFZ, 04318 Leipzig, Germany; Department of Computation Biology, Helmholtz Centre for Environmental Research – UFZ, 04318 Leipzig, Germany; Department of Computer Science, Faculty of Mathematics and Computer Science, University of Leipzig, 04109 Leipzig, Germany; Department of Computation Biology, Helmholtz Centre for Environmental Research – UFZ, 04318 Leipzig, Germany

## Abstract

**Summary:**

Sophisticated approaches for the *in silico* prediction of toxicity are required to support the risk assessment of chemicals. The number of chemicals on the global chemical market and the speed of chemical innovation stand in massive contrast to the capacity for regularizing chemical use. We recently proved our ready-to-use application deepFPlearn as a suitable approach for this task. Here, we present its extension deepFPlearn${}^{+}$ incorporating (i) a graph neural network to feed our AI with a more sophisticated molecular structure representation and (ii) alternative train-test splitting strategies that involve scaffold structures and the molecular weights of chemicals. We show that the GNNs outperform the previous model substantially and that our models can generalize on unseen data even with a more robust and challenging test set. Therefore, we highly recommend the application of deepFPlearn${}^{+}$ on the chemical inventory to prioritize chemicals for experimental testing or any chemical subset of interest in monitoring studies.

**Availability and implementation:**

The software is compatible with python 3.6 or higher, and the source code can be found on our GitHub repository: https://github.com/yigbt/deepFPlearn. The data underlying this article are available in Zenodo, and can be accessed with the link below: https://zenodo.org/record/8146252. Detailed installation guides via Docker, Singularity, and Conda are provided within the repository for operability across all operating systems.

Key messages

- Graph neural networks are superior in providing molecular structure information to downstream tasks.
- Scaffold splitting ensures models generalize better to unseen molecular structures providing more realistic performance estimates.
- deepFPlearn ${}^{+}$ enhances the predictive power and feasibility of deep learning in toxicology for smart chemical prioritization and sustainable design.

## 1 Introduction

Recently, we developed the ready-to-use and stand-alone program deepFPlearn that predicts the association between chemical structures and effects on the gene/pathway level using a combined deep learning approach (Schor *et al.* 2022). We achieved high accuracy using a deep autoencoder (AE) to reduce features and a feed-forward neural network to predict whether the input chemical interacts with the nuclear receptors involved in endocrine disruption. Our pretraining strategy allowed for capturing a vast range of molecular structures. In addition, deepFPlearn classifies chemicals quickly and can be customized. Our tool significantly aids in the systematic *in silico* study of chemicals, and can handle the vast, constantly growing chemical universe.

deepFPlearn uses binary topological fingerprints to encode the molecular structure of chemicals, which show the absence or presence of certain substructures or atomic configurations by ones and zeros respectively, in a fixed-size binary vector. deepFPlearn${}^{+}$ now can also use molecular graphs to encode the structure of chemicals preserving the full connectivity information which is not fully captured in binary fingerprints, at the expense of lost chirality information. Graph neural networks can process such graph-based information.

A graph neural network (GNN) is an artificial neural network that operates on graph data (Scarselli *et al.* 2009). A graph G=(V,E) comprises a set of nodes *V* and a list of edges *E* that encodes the nodes’ relations. In molecular structure graphs, nodes represent atoms, and covalent chemical bonds form the edges. More attributes, like the type of atom or direction/type of the bond, can be stored in *V* and *E*. A GNN then forms an optimizable transformation on all graph attributes that preserve graph symmetries. One approach for training a GNN is using message passing (MP), which involves disseminating information stored in nodes and edges as a cumulative message throughout the network to arrive at a prediction. In a D-MPNN, the message is associated with directed edges to avoid unnecessary loops in the message-passing trajectory. See Yang *et al.* (2019) for the detailed algorithm.

Substructures form functional entities—scaffolds—and different scaffold combinations result in different properties and modes of interactions with other molecules. A *chemical scaffold* is a structure that is shared among a group of molecules, which are then likely to have a similar relationship with targets (Katritzky *et al.* 2000). The cheminformatic software package RDKit (Landrum 2006) implements algorithms from Bemis and Murcko (1996) that generate chemical scaffolds from their 2D representation. Typically, machine learning methods require datasets to be split into train and test sets. While trained on the first, the model is evaluated on the latter to investigate how well the trained model can generalize on unseen data. A random split creates a similar distribution of classes in the train and test set. This is only sometimes best for chemical structures since both sets contain similar structures, leading to an overestimation of the model’s prediction performance. Scaffold splitting separates structurally different molecules into different subsets, providing a more significant challenge for learning algorithms than the random split. Splitting based on molecular weight divides datasets into training and test sets by considering the size of the molecules. This approach aims to ensure that molecules of different sizes are represented in both sets, reflecting the diversity of molecular scales.

The idea to reach out beyond random split is that the increased differences between training and test sets form a more robust test of the model’s generalizability (Wu et al. 2018).

Here, we present deepFPlearn${}^{+}$ - an extension of deepFPlearn incorporating graph neural networks to predict the association of an entire graph (one chemical) with selected nuclear receptors involved in endocrine disruption and alternative train-test data splitting strategies.

## 2 deepFPlearn${}^{+}$

**Figure 1.**
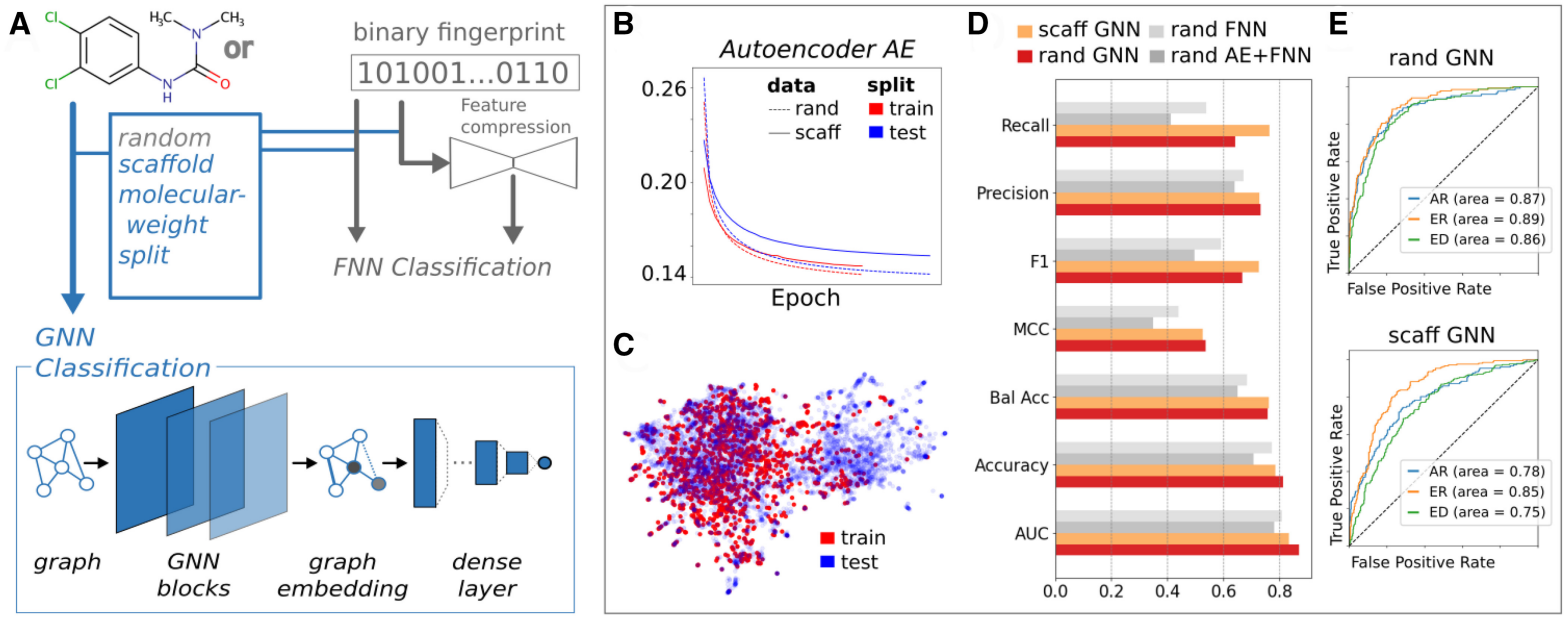
(A) DL framework with deepFPlearn${}^{+}$ extensions indicated in blue and existing parts in gray. (B) History of AE mean squared error for random and scaffold split of the train data. (C) Uniform Manifold Approximation and Projection (UMAP) embedding of the scaffold-split with a noticeable distribution-shift between train and test data. (D) Comparison of all collected metrics across all model architectures for the ED (endocrine disruptor) target using the best fold for each model. (E) ROC-AUC of random and scaffold split of GNN for three targets, AR (androgen receptor), ER (estrogen receptor-alpha), and ED (endocrine disruptor).

We extended our original deepFPlearn deep learning framework by a D-MPNN graph neural network and two alternative train test data splitting strategies, based on structural scaffolds and the molecular weight of the chemicals (see Fig. 1A). We trained deepFPlearn on the same data as in Schor *et al.* (2022) and compared the model performances for different setups. In particular, we performed a 5-fold cross-validation for training, employed Weights & Biases (www.wandb.com) for hyperparameter tuning, and used the small but labeled dataset *S* [extracted from the supplemental material of Sun *et al.* (2019)] containing 7248 compounds for classification and the huge unlabeled dataset *D* [extracted from the CompTox Chemistry Dashboard (Williams *et al.* 2017, accessed on 13 July 2020)] containing 719 996 compounds for pretraining and feature compression. When we enabled scaffold split for pretraining, the average validation loss of the AEs increased slightly from 0.1386 (random split) to 0.1547 (Fig. 1B), which we expected since the molecular structures differ substantially in the train and the test data (Fig. 1C). With enabled molecular weight split for pretraining, the average validation loss of the AEs increased substantially to 0.2266 (Supplementary Fig. S1). See supplementary Fig. S1 for the training performances and UMAP visualizations of the training data for all three cases. Further, the validation loss diverges considerably from the training loss. We expected this even more since this approach ensures that the differences between molecules of the train and test datasets are much higher. Consequently, the potential of over- and underfitting specific molecular weight ranges grows dramatically, and the model’s generalizability declines. The GNN (with random split) substantially outperforms the fingerprint classification models (Schor *et al.* 2022). We reached binary accuracy values of 0.87, 0.84 and 0.81 and ROC-AUC values of 0.87, 0.89, 0.86 for androgen receptor (AR), estrogen receptor (ER), and endocrine disruption (ED), respectively. Enabling scaffold split slightly reduced the model’s performance. And still, we reached higher values with the GNN than we did when using the molecular fingerprint as input and the traditional feed-forward neural network (see Fig. 1D and E). See the supplementary material for detailed performance comparisons showing all combinations of split strategies, model architectures, and classification targets.

We used the trained GNN model for ER to predict the association between all substances from the *D* dataset, which have no association with ER in our training data. Exemplary, we found Hexylresorcinol, Quinoxyfen, and Clofoctol among the top positive predictions, with a probability higher than 80%. The CompTox dashboard provides bioassay information for all three substances, indicating an association with ER. Hexylresorcinol is a substituted dihydroxybenzene with antiseptic, anthelmintic, and local anesthetic properties, contained in topical applications for minor skin infections and oral solutions or throat lozenges for pain relief and first-aid antiseptic (Chaudhuri and Chaudhuri, Matthews et al. 2020) Quinoxyfen is a fungicide used mainly to control *Erysiphe graminis*—powdery mildew in cereals. The EFSA regulated the maximum amount of this substance based on bioactivity and toxicity studies in December 2020 EFS. Clofoctol is a bacteriostatic antibiotic for treating upper and lower respiratory tract infections. The CompTox Chemistry Dashboard reports bioactivity with ER in the ToxCast summary reports.

## 3 Conclusion

In the deepFPlearn architecture, FFNNs had established a sophisticated baseline for performance, whereas AEs facilitated a pretrained model attuned to a vast array of chemical structures from unlabeled datasets. Although these methods performed good enough, we substantially improved the performance of deepFPlearn by tuning the input data representation and, subsequently, the model’s architecture toward a graph neural network. To address the generalizability assessment of GNNs that lack pretraining, we introduced more challenging train-test splitting strategies of scaffold and molecular weight split and compared the model performances to the original random split approach. Scaffold split was most feasible since it provides a more realistic test set regarding the application domain of deepFPlearn${}^{+}$. It tests the model’s generalizability by separating structurally different molecules into separate subsets.

With deepFPlearn${}^{+}$   we increased the predictive power and the feasibility of the overall beneficial deep-learning approach in predictive toxicology. We could confirm our predictions with current toxicity knowledge from the latest ToxCast release, suggesting that there are more exciting chemicals among them that should be considered in subsequent wet lab and *in silico* analyses. In ongoing data analyses, we dive deeper into the biological backgrounds of our predictions and integrate explainable AI into our framework to better understand and explain our models’ decisions. We promote deepFPlearn${}^{+}$ś application on the chemical inventory and custom subsets of substances to prioritize chemicals for experimental testing, assist in the smart selection of chemicals for monitoring and contribute to the sustainable design of future chemicals.

## Supplementary Material

btad713_Supplementary_Data
