# Supplementary material for: deepFPlearn +: enhancing toxicity prediction across the chemical universe using graph neural networks
Source: Bioinformatics. 2023 Nov 27;39(12):btad713. doi: 10.1093/bioinformatics/btad713 (PMC10724847; doi:10.1093/bioinformatics/btad713)
Supplement: btad713_Supplementary_Data [file btad713_supplementary_data.pdf]

Bioinformatics, 2023, 1–5
doi: DOI HERE
Advance Access Publication Date: Day Month Year
Application note

`deepFPlearn`$^{+}$: Enhancing Toxicity Prediction Across the Chemical Universe Using Graph Neural Networks

Kyriakos Soulios [1,3], Patrick Scheibe [2], Matthias Bernt [1], Jörg Hackermüller [1,3] and Jana Schor [1,*]

*Corresponding author. jana.schor@ufz.de

FOR PUBLISHER ONLY Received on Date Month Year; revised on Date Month Year; accepted on Date Month Year

Abstract

Summary: Sophisticated approaches for the *in-silico* prediction of toxicity are required to support the risk assessment of chemicals. The number of chemicals on the global chemical market and the speed of chemical innovation stand in massive contrast to the capacity for regularizing chemical use. We recently proved our ready-to-use application as a suitable approach for this task. Here, we present its extension `deepFPlearn`$^{+}$ incorporating i) a graph neural network to feed our AI with a more sophisticated molecular structure representation and ii) alternative train-test splitting strategies that involve scaffold structures and the molecular weights of chemicals. We show that the GNNs outperform the previous model substantially and that our models can generalize on unseen data even with a more robust and challenging test set. Therefore, we highly recommend the application of `deepFPlearn`$^{+}$ on the chemical inventory to prioritize chemicals for experimental testing or any chemical subset of interest in monitoring studies.
Availability and Implementation: The software is compatible with python 3.6 or higher, and the source code can be found on our GitHub repository: `https://github.com/yigbt/deepFPlearn`. A complete data and models archive is also available on Zenodo: `https://zenodo.org/record/8146252`. Detailed installation guides via Docker, Singularity, and Conda are provided within the repository for operability across all operating systems.
Contact: jana.schor@ufz.de
Supplementary information: The supplementary material is provided in the submission.

Key words: deep learning, graph neural network, scaffold split, chemical structure, chemical-effect associations

Data splitting strategies

Here we present the three splitting strategies offered in our software.

- **Random split**: Samples are randomly assigned to the training and test sets, assuming independent and identically distributed data. This strategy creates train and test sets with similar distributions, making the learning task easier.
- **Scaffold balanced split**: Molecules are grouped based on their core scaffold which is calculated by RDKit. Within each scaffold group, molecules are randomly assigned to the training or test set. This ensures that structurally similar molecules are present in only one of the sets, allowing evaluation of the model's generalization to new chemical scaffolds. The balance ensures that not only the scaffold groups with the fewest chemicals are assigned to the test set.
- **Molecular weight split**: The dataset is divided based on the molecular weight of the compounds. Molecules are separated into different sets according to their size. This strategy helps assess the model's performance on larger molecules since the chemistry rules govern both the small and the large molecules.

Each splitting strategy serves a specific purpose in evaluating machine learning models for molecular tasks. Random split provides a baseline evaluation, scaffold split evaluates generalization to new scaffolds, and molecular weight split assesses performance across different molecule sizes. The choice of splitting strategy depends on the specific goals of the study and the characteristics of the dataset.

Pretraining with autoencoders

The goal of any autoencoder is to reconstruct its input. In our case, the autoencoder aims to capture some 'general chemistry

© The Author 2023. Published by Oxford University Press. All rights reserved. For permissions, please e-mail: journals.permissions@oup.com

knowledge' from the large unlabeled dataset by compressing and reconstructing the molecular fingerprints. Usually during the autoencoder training(pretraining), it is common practice to use split ratios that favor more the training set to exploit most of the available data in a random fashion. Randomly splitting the data usually creates train and test sets with similar distributions and therefore making the reconstruction an easier task. So, we explored the use of different data splitting methods at the autoencoder level. We view data splitting as modifying the task of the autoencoder. It is again reconstruction but now the mew split method makes the reconstruction harder. At first, we used 0.8/0.2 as our train/validation ratio and randomly splitting the distribution of the training and test data is almost overlapping as assumed, and thus the test loss achieved is low. By using scaffold split, we start noticing a small drift between the distributions and a slight increase in the validation loss. The data are so much and the variety of scaffolds low though that the drift is barely noticeable, and this is reflected on the proximity of the validation loss to the one of the random split. By splitting the data based on molecular weight, the drift is easily observable and hence the validation loss score is worse as shown in the figure 1.

Downstream tasks

Using data splitting methods, such as scaffold split or molecular weight split, on downstream tasks in machine learning for molecular applications is a common practice and provides a more robust way to evaluate the model's performance. These splitting methods ensure that the model is tested on unseen chemical scaffolds or molecule sizes, respectively, which reflects its ability to generalize to new and diverse molecular structures. However, combining different splitting methods at different levels, such as using scaffold split during pretraining and molecular weight split during fine-tuning, can potentially offer additional benefits. This combined approach exposes the model to a wider range of structural variations and size distributions, leading to a more comprehensive evaluation of its capabilities. By leveraging multiple splitting strategies, we can gain deeper insights into the model's performance and uncover any potential biases or limitations. This holistic approach helps enhance the model's robustness and enables a more accurate assessment of its performance on downstream tasks. The fig:loss,fig:AUCs support these assumptions as we can see the diagonal (where the data splitting is the same on both levels) of the ROC-AUC plots rarely achieving the best performance.

Graph neural networks

To understand the performance of the graph neural networks we can observe their training histories in the following plots 4 - 5. We notice that under 15 epochs the GNN starts to overfit. The bigger the drift between the training and validation distribution, the faster the overfitting. We can notice a significant improvement over the AUC across all data splitting methods compared to the fingerprint based methods. We can also notice the differences of the different splitting methods by looking at the confusion matrices in the figure6 below. In toxicity prediction, we do not want to misidentify any potentially toxic chemicals and thus we want to limit the false negatives or the top right box of every matrix to be the lightest possible and consequently recall the highest.

A final comparison between the best fingerprint based methods and the GNNs can be highlighted from the barplots 7 below.

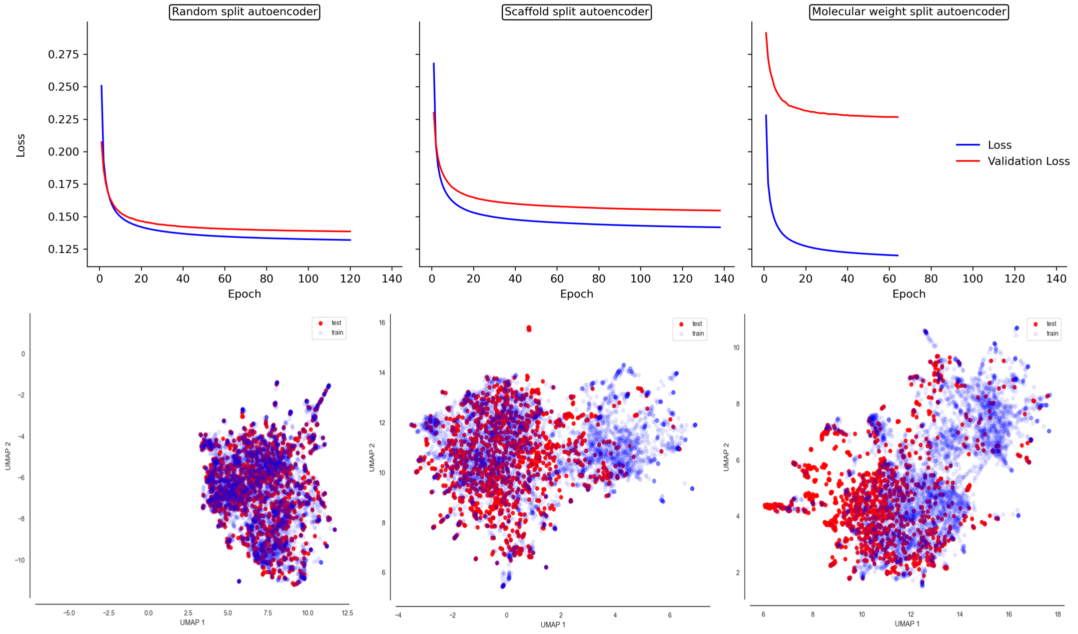

Fig. 1: On the first row we can see the autoencoder histories using the three ways of splitting. The blue line represents the training loss while the red represents the validation loss. The UMAPs are based on the compressed version of the fingerprints after the autoencoder training. They are produced on samples of 10 000 datapoints from the 800 000 initial dataset. The drift between training and validation set bacomes noticeable as the splitting strategy changes. While in random split the distributions between the sets are similar, the scaffold makes them diverging and the molecular weight almost slightly-overlapping.

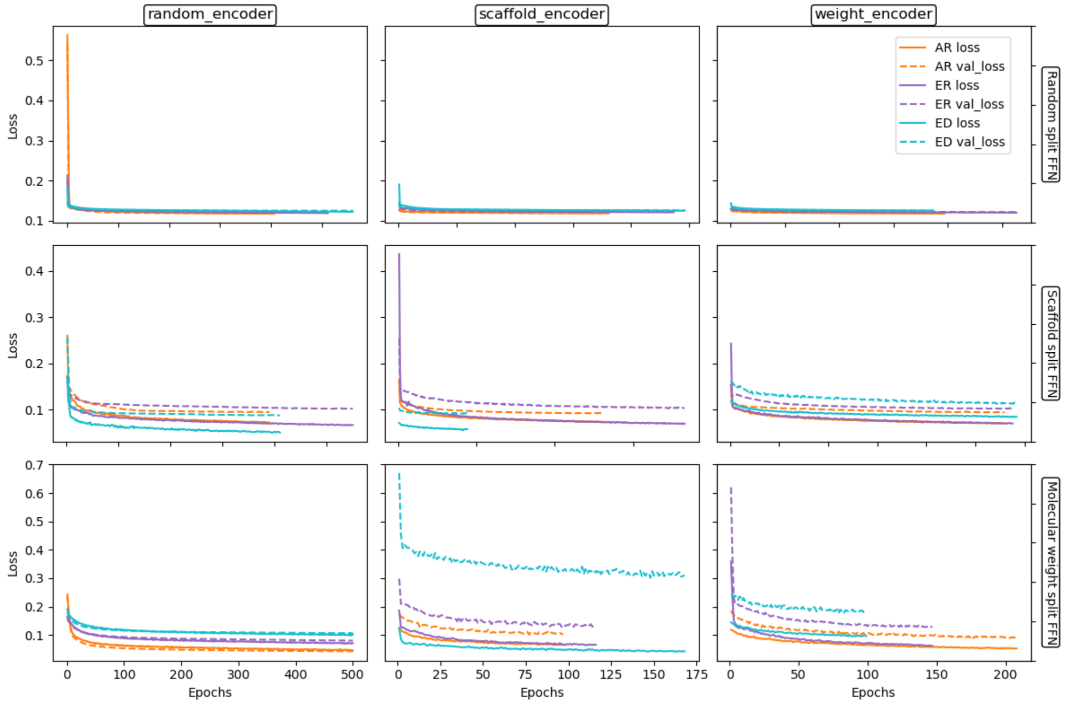

Fig. 2: Training histories of all possible combinations of the classification models stratified by split type on the autoencoder and the FFN level for targets AR, ER, and ED. Dashed lines (–) represent the validation set while the solid ones (-) mark the training set. Each target is color coded, orange for AR, Purple for ER and turquoise for ED On the x-axis of the image we notice different split strategies on the autoencoder level and on the y-axis on the feed forward model.

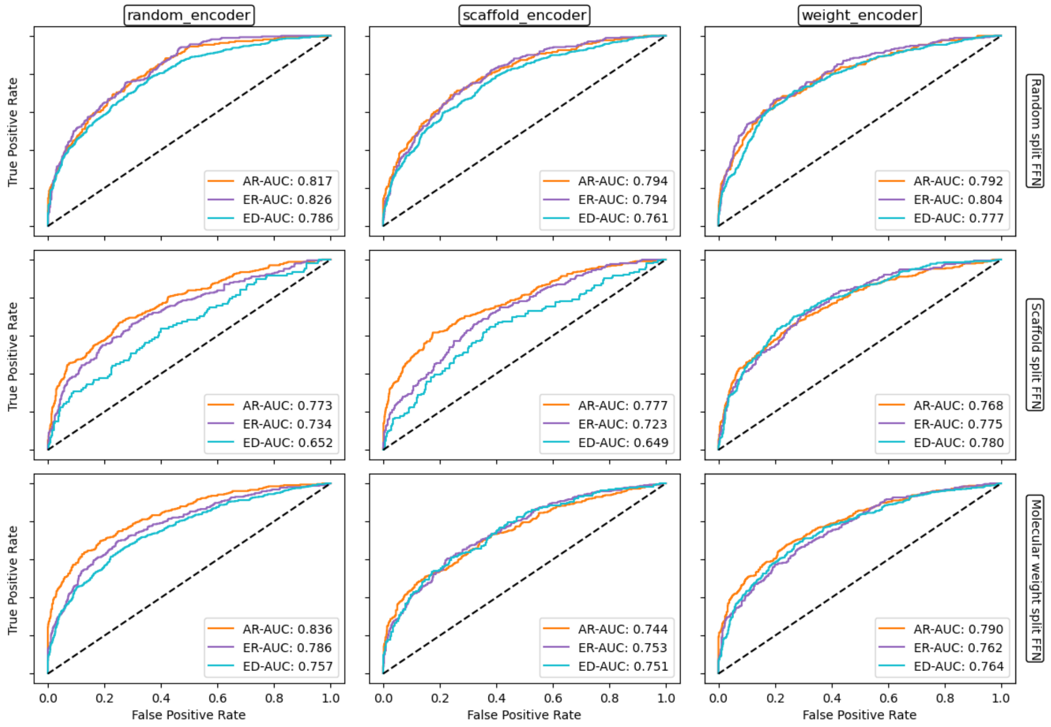

Fig. 3: AUC plots of all possible combinations of the classification models stratified by split type on the autoencoder and the FFN level for targets AR, ER, and ED.

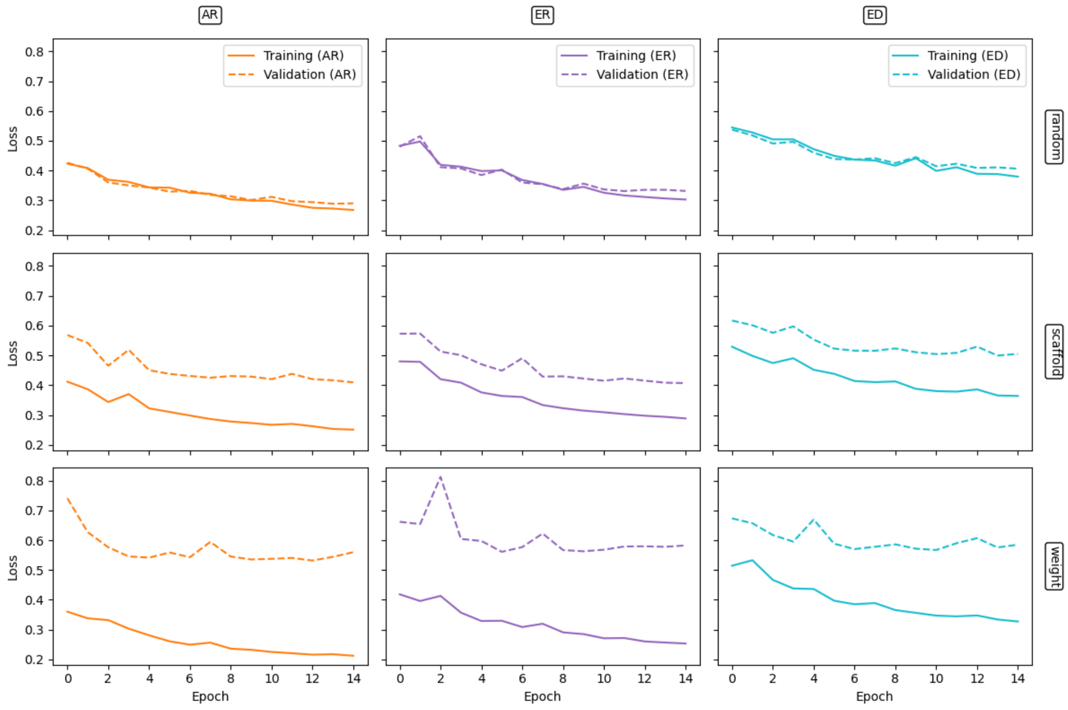

Fig. 4: Training histories of the GNNs stratified by data split type for targets AR, ER, and ED. Dashed lines (–) represent the validation set while the solid ones (-) mark the training set. Only the random split produces similar performance on training and validation set.

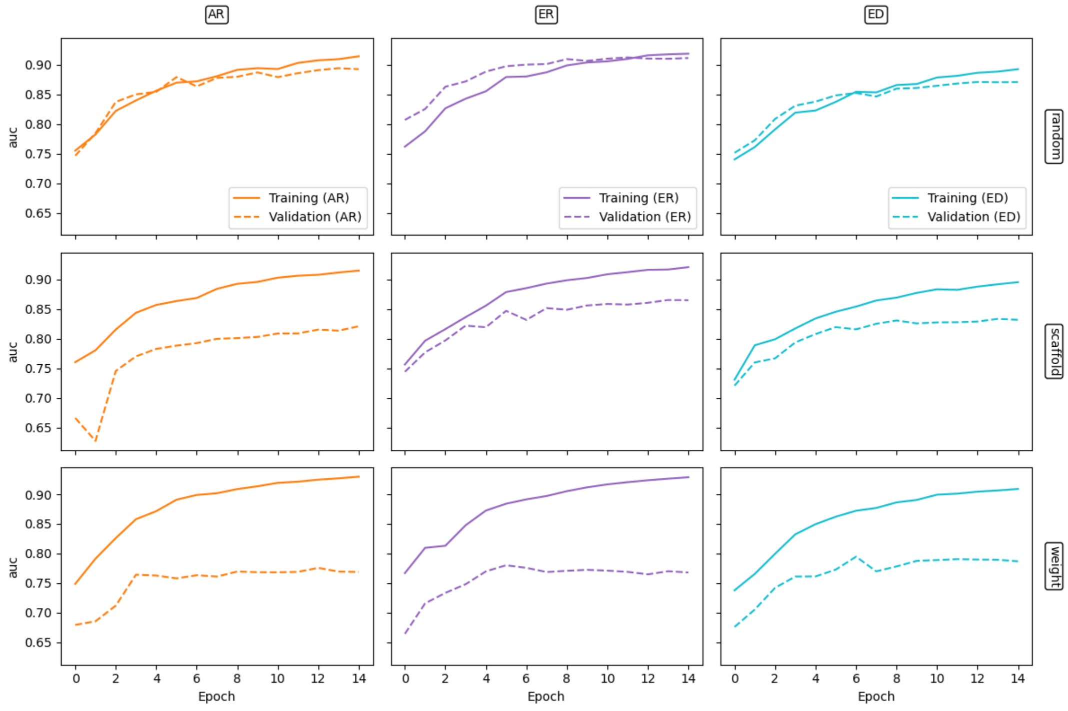

Fig. 5: AUC plots of all combinations of the GNNs stratified by data split type for targets AR, ER, and ED. Dashed lines (–) represent the validation set while the solid ones (-) mark the training set. In scaffold and molecular weight split there is a noticeable difference between the training and validation metrics due to the distribution shift.

We can notice that the GNNs outperform the fingerprint based networks across all metrics when random split is used. Even when more difficult splitting methods are used the GNNs can offer competitive results.

Estrogenic risk assessment

Our predictive model has achieved a remarkable feat by accurately forecasting the estrogenic activity of Hexylresorcinol, Quinoxyfen, and Clofoctol, despite not being initially trained on these specific compounds. As such, our model could serve as a valuable tool for guiding toxicity assessments and, in the foreseeable future, may even replace traditional methods for a more efficient and comprehensive evaluation of chemical safety.

Hexylresorcinol

Hexylresorcinol presents a unique case of potential risk due to its widespread use in everyday products like throat lozenges and cosmeticsChaudhuri and Chaudhuri, Matthews et al.. While it may not bioaccumulate in the traditional environmental sense, the frequent and varied routes of exposure—oral consumption through lozenges and dermal application through cosmetics—could lead to a form of 'lifestyle accumulation' in humans. Given its predicted estrogenic activity, this raises significant concerns about its long-term impact on hormonal balance and reproductive health. The compound's ubiquity in consumer products necessitates an immediate and thorough regulatory review to assess its safety from an endocrine-disrupting perspective.

Quinoxyfen

Primarily used as a pesticide-biocide in agricultural settings, Quinoxyfen aims to control fungal diseases like powdery mildew in crops. The predicted values for its environmental fate corroborate this concernArena et al., qui. Its high potential for bioaccumulation and bioconcentration in aquatic organisms could lead to biomagnification of the food chain, affecting humans who consume contaminated water or fish. While it may degrade relatively quickly, its persistence in soil and water bodies could have far-reaching implications for both environmental and human health. Given its predicted estrogenic activity and its complex risk profile, Quinoxyfen warrants re-evaluation and possible regulatory action to mitigate its potential risks.

Clofoctol

Clofoctol, a bacteriostatic antibiotic marketed in France and Italy since 2005Bailly and Vergoten [2021], exemplifies the regulatory gaps in assessing the endocrine-disrupting potential of pharmaceuticals. It was also discovered to be an antiviral against SARS-CoV-2 or even a potential prostate cancer inhibitorBelouzard et al., Wang et al.. Despite its long-standing use for treating bacterial infections, its estrogenic activity has not been studied, owing to the lack of regulatory mandates for such testing. This oversight calls for an immediate overhaul in pharmaceutical regulations to include mandatory endocrine-disrupting activity assessments for all drugs, especially those that have been on the market for an extended period.

In summary, Hexylresorcinol, Quinoxyfen, and Clofoctol each present unique challenges that highlight the urgent need for more comprehensive regulatory frameworks. These frameworks should not only focus on the primary intended effects of these compounds but also consider their potential as endocrine disruptors, thereby safeguarding both environmental and human health.

References

Quinoxyfen - chemical details.

M. Arena, D. Auteri, S. Barmaz, G. Bellisai, A. Brancato, D. Brocca, L. Bura, H. Byers, A. Chiusolo, D. C. Marques, F. Crivellente, C. D. Lentdecker, M. Egsmose, Z. Erdos, G. Fait, L. Ferreira, M. Goumenou, L. Greco, A. Ippolito, F. Istace, S. Jarrah, D. Kardassi, R. Leuschner, C. Lythgo, J. O. Magrans, P. Medina, I. Miron, T. Molnar, A. Nougadere, L. Padovani, J. M. P. Morte, R. Pedersen, H. Reich, A. Sacchi, M. Santos, R. Serafimova, R. Sharp, A. Stanek, F. Streissl, J. Sturma, C. Szentes, J. Tarazona, A. Terron, A. Theobald, B. Vagenende, A. Verani, and L. Villamar-Bouza. Peer review of the targeted hazard assessment of the pesticide active substance quinoxyfen. *EFSA Journal*, 16:e05085, 1 . ISSN 1831-4732. doi: 10.2903/J.EFSA.2018.5085.

C. Bailly and G. Vergoten. A new horizon for the old antibacterial drug clofoctol. *Drug Discovery Today*, 26:1302–1310, 5 2021. ISSN 1359-6446. doi: 10.1016/J.DRUDIS.2021.02.004.

S. Belouzard, A. Machelart, V. Sencio, T. Vausselin, E. Hoffmann, N. Deboosere, Y. Rouillé, L. Desmarets, K. Séron, A. Danneels, C. Robil, L. Belloy, C. Moreau, C. Piveteau, A. Biela, A. Vandeputte, S. Heumel, L. Deruyter, J. Dumont, F. Leroux, I. Engelmann, E. K. Alidjinou, D. Hober, P. Brodin, T. Beghyn, F. Trottein, B. Deprez, and J. Dubuisson. Clofoctol inhibits sars-cov-2 replication and reduces lung pathology in mice. *PLOS Pathogens*, 18:e1010498, 5 . ISSN 1553-7374. doi: 10.1371/JOURNAL.PPAT.1010498.

R. Chaudhuri and R. K. Chaudhuri. Hexylresorcinol: Providing skin benefits by modulating multiple molecular targets photostability view project skin hydration and barrier building view project hexylresorcinol: Providing skin benefits by modulating multiple molecular targets.

D. Matthews, O. Adegoke, and A. Shephard. Bactericidal activity of hexylresorcinol lozenges against oropharyngeal organisms associated with acute sore throat. *BMC Research Notes*, 13:1–4, 2 . ISSN 17560500. doi: 10.1186/S13104-020-04954-1/FIGURES/1.

M. Wang, J. S. Shim, R.-J. Li, Y. Dang, Q. He, M. Das, and J. O. Liu. Identification of an old antibiotic clofoctol as a novel activator of unfolded protein response pathways and an inhibitor of prostate cancer correspondence. *Journal of Pharmacology*, 171:4478–4489. doi: 10.1111/bph.12800.

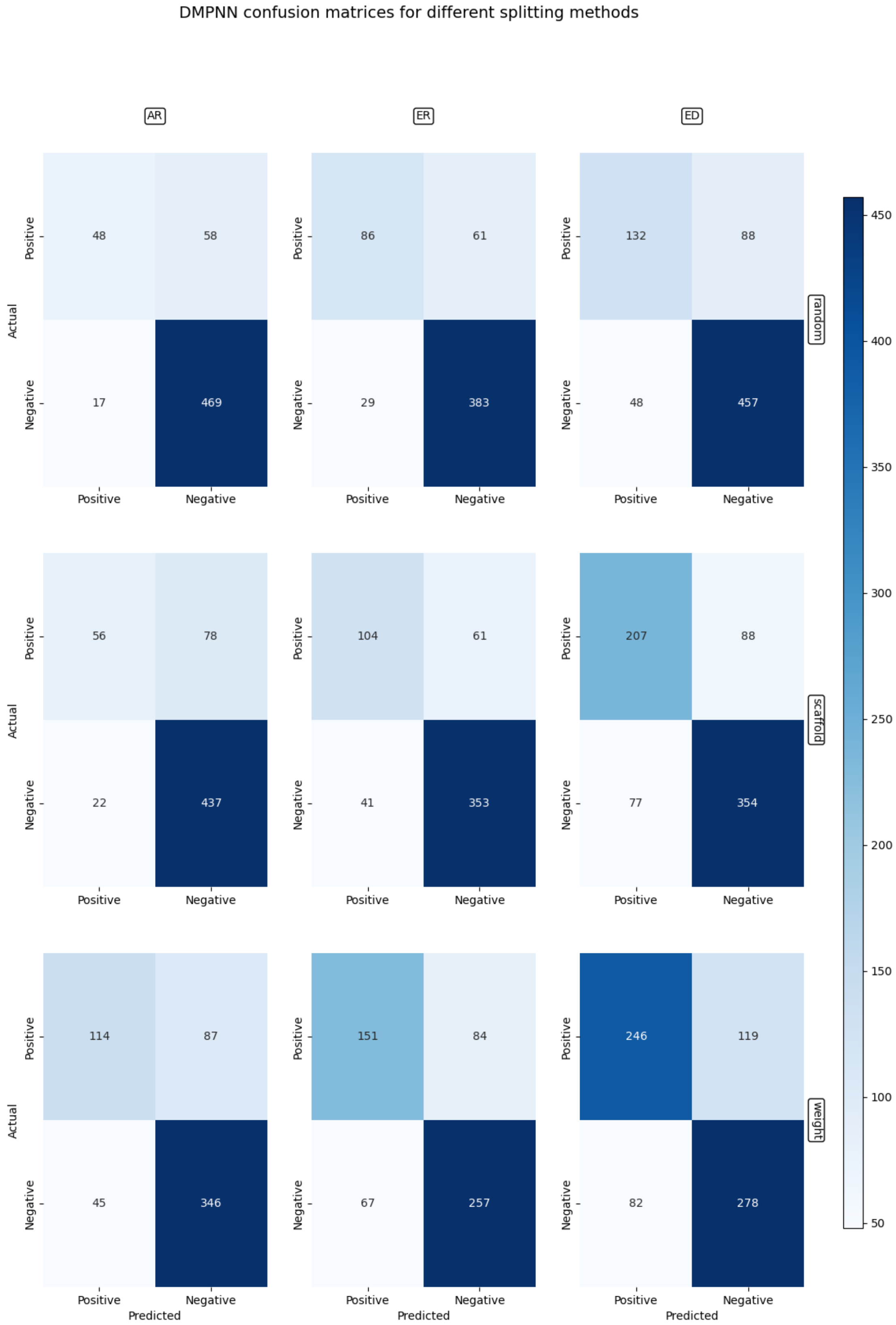

Fig. 6: GNN confusion matrices stratified by target and splitting method. In the top left corner we can see the true positives and on the bottom left the true negatives. The darker the shade the higher the absolute values. In these two quarters, the darker means better. In the top right corner and bottom left, the reverse is true.

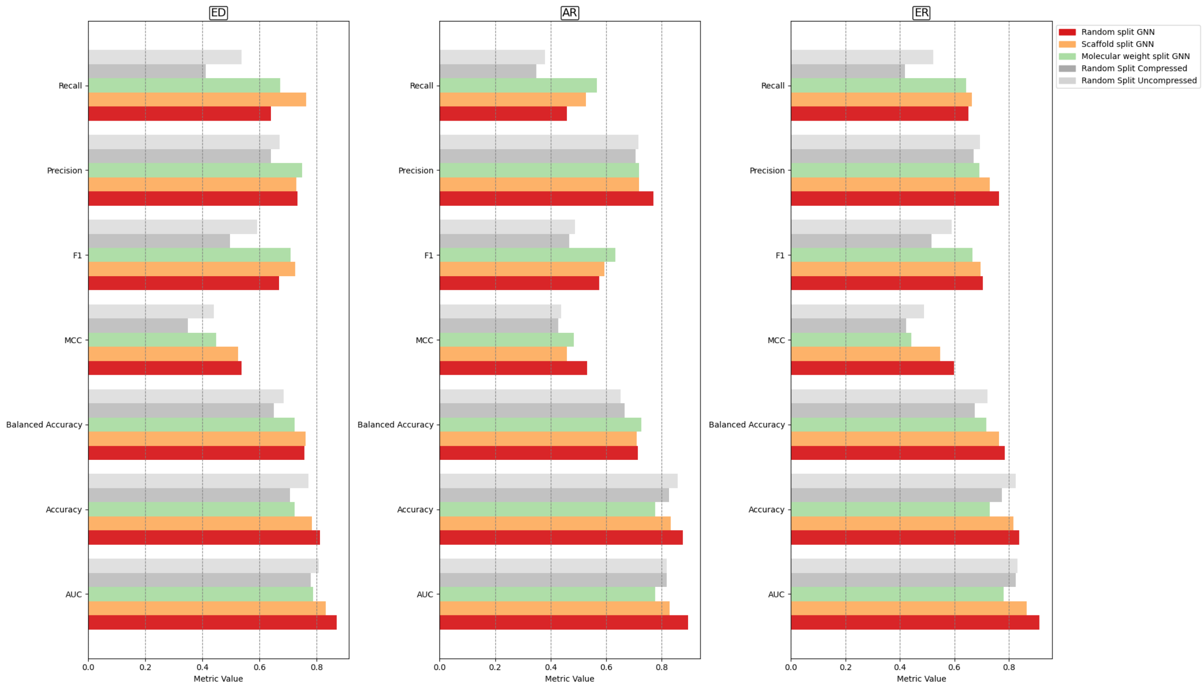

Fig. 7: Metrics barplot comparison between GNNs and fingerprint based methods, stratified by target. The random splitted models using the fingerprints in compressed and uncompressed versions were chosen to be plotted and are shown in greyish colors. All the versions of the GNN model are color coded, red for random split, orange for scaffold aand green for molecular weight. The metrics that take into account the imbalance are almost always better in the case of GNN models.
